# Perceived principals’ aesthetic leadership and teacher professional development: the roles of aesthetic experience and psychological empowerment among Chinese high school teachers

**DOI:** 10.3389/fpsyg.2026.1846303

**Published:** 2026-09-10

**Authors:** Zehe Yin, Yuancheng Chang, Fengde Qian, Weixin Lin, Kexiu Fang

**Affiliations:** 1 College of Design, Hainan Vocational University of Science and Technology, Haikou, China; 2 Chinese International College, Dhurakij Pundit University, Bangkok, Thailand; 3 School of Design, Nanjing Tech University, Nanjing, China

**Keywords:** aesthetic experience, high school teachers, principals’ aesthetic leadership, psychological empowerment, teacher professional development

## Abstract

Aesthetic leadership emphasizes the experiential dimensions of leadership processes, including sensory engagement, emotion, embodied presence, and symbolic expression. Its focus differs from that of transformational leadership, which primarily emphasizes visionary inspiration, and from school climate research, which centers on shared perceptions of the organizational environment. However, although emerging evidence has linked perceived principals’ aesthetic leadership with teacher professional development in Chinese high schools, the experiential processes and psychological boundary conditions underlying this association remain insufficiently understood. In China’s highly structured school governance context, where principals play a central role in interpreting policy and organizing teachers’ work, aesthetic leadership may be especially relevant because it addresses how leadership is experienced emotionally and symbolically in everyday school life. Using cross-sectional self-report questionnaire data from 611 high school teachers in Hainan Province, China, this study examined the association between perceived principals’ aesthetic leadership (AL) and teacher professional development (TPD), the statistical indirect role of aesthetic experience (AE), and the conditional role of psychological empowerment (PE). AMOS was used to examine the latent-variable mediation relationship, while SPSS PROCESS Model 59 was employed to estimate interaction terms and conditional indirect effects within a unified observed-variable framework. AL was significantly and positively associated with both TPD and AE, and AE was positively associated with TPD. The statistical indirect association between AL and TPD through AE was significant. PE did not significantly moderate the AL–AE association. However, the AL × PE interaction in predicting TPD was significantly positive, whereas the AE × PE interaction was significantly negative. As PE increased, the conditional direct association between AL and TPD became stronger, whereas the AE–TPD association and the conditional indirect association became weaker. For school principals and teacher professional development organizers, the findings suggest combining transparent management with meaning-centered communication and autonomy-supportive professional learning activities. Given the single-wave self-report design, these findings represent statistical associations and cannot establish temporal ordering or causal mechanisms.

## 1 Introduction

In recent years, aesthetic leadership has emerged as a new perspective in educational leadership research. Aesthetic leadership (AL) focuses on how leaders use sensory, symbolic, and emotional means to cultivate an “aesthetic atmosphere” within organizations, with particular emphasis on followers’ subjective experiences and meaning-making (Hansen et al., 2007; Howard, 1996). Unlike transformational leadership, which emphasizes vision and goal orientation, and school climate research, which focuses on shared perceptions of organizational norms and practices, aesthetic leadership highlights how leaders’ cultural symbolism, embodied presence, and emotional expression may shape organizational members’ experiences and interpretations of leadership (Bathurst and Monin, 2010; Hansen et al., 2007; Ropo and Sauer, 2008). Existing research suggests that aesthetic perceptions may strengthen followers’ evaluations of leaders’ charisma and authenticity and may be associated with a more creative and supportive organizational climate (Duhamel et al., 2023; Hansen et al., 2007). China’s school governance system has traditionally been characterized by relatively centralized policy direction and hierarchical authority, although educational reforms have introduced varying degrees of local and school-level autonomy. Within this structure, principals occupy a pivotal position in translating system-level policy requirements into school-level priorities and organizing teachers’ everyday work (OECD, 2020). Aesthetic leadership is therefore particularly relevant in this context because it draws attention to how teachers experience principals’ symbolic communication, emotional tone, and cultural practices within a formally structured organizational environment (Hansen et al., 2007; Scott and Myers, 2010). However, although emerging evidence has begun to link principals’ aesthetic leadership with teacher professional development in Chinese school contexts, the psychological processes and boundary conditions underlying this association remain insufficiently understood. Against this institutional backdrop, the present study investigates the association between teachers’ perceptions of principals’ aesthetic leadership and their professional development.

Teacher professional development (TPD) is commonly conceptualized as a dynamic process through which teachers continuously learn, reflect, and improve their knowledge, competencies, beliefs, and practices. Its quality is important not only for teachers’ individual growth but also for instructional quality and school improvement (Ingvarson et al., 2005; Sancar et al., 2021). Organizational research has also shown that managerial favoritism and nepotism are associated with more negative evaluations of the organizational climate (Vveinhardt and Bendaraviciene, 2022; Vveinhardt and Jakucionyte, 2023). Accordingly, aesthetic leadership should not be treated as an isolated leadership attribute detached from the broader school context. Rather, it should be understood within a school management environment characterized by fairness, respect, safety, and transparency. How school leaders are associated with teachers’ sustained professional growth through cultural development, emotional support, and professional encouragement has therefore become an important issue in educational management research (Hallinger and Heck, 2010; Keung et al., 2020; Noonan, 2019; Anderson et al., 2022). Against this background, aesthetic leadership, as a leadership paradigm that emphasizes sensory engagement, emotional resonance, symbolic expression, and the shaping of the organizational atmosphere, has attracted increasing attention in organizational and educational research (Howard, 1996; Hansen et al., 2007; Bathurst and Monin, 2010).

Unlike traditional leadership approaches that emphasize institutional control and rational decision-making, aesthetic leadership places greater emphasis on how leaders use language, behavior, the physical environment, and embodied presence to shape organizational members’ identification, imagination, and interpretations of organizational life. In school settings, principals’ aesthetic leadership practices may therefore represent an important organizational resource associated with teacher professional development (Ropo and Sauer, 2008; Bates and Stanley, 2006).

Although leadership is widely regarded as an important factor associated with teacher professional development, existing research has focused primarily on transformational, instructional, and collaborative leadership, with comparatively limited attention to aesthetic leadership in school settings (Hallinger and Heck, 2010; Liu and Hallinger, 2018; Hsieh et al., 2021). A related study by Yin and Chang (2025) examined the association between high school teachers’ perceptions of principals’ aesthetic leadership and teacher professional development in Hainan Province, with work autonomy as a mediating variable. Building on that study, the present research addresses a different psychological process and boundary condition. Specifically, rather than re-examining the work-autonomy mediation pathway, the present study investigates aesthetic experience as an experiential statistical intermediary and psychological empowerment as a moderator of the associations among perceived principal aesthetic leadership, aesthetic experience, and teacher professional development. Thus, the present study extends the earlier work by shifting the explanatory focus from work autonomy to aesthetic experience and by examining whether these associations vary across levels of psychological empowerment.

More broadly, the existing literature has not yet adequately explained the experiential processes and boundary conditions associated with aesthetic leadership and teacher professional development. Leadership perceptions do not necessarily correspond uniformly to professional development outcomes; an important consideration is how teachers perceive, interpret, and internalize the emotional and cultural meanings communicated by principals (Scott and Myers, 2010). Aesthetic experience (AE) may represent an important experiential link between aesthetic leadership and teacher professional development because it encompasses engagement, emotional resonance, and meaning-making in educational settings, which may in turn be associated with teachers’ professional identity and professional actions (Hobbs, 2012; Hobbs and Kelly, 2017). Psychological empowerment (PE), meanwhile, reflects individuals’ perceptions of meaning, competence, self-determination, and impact at work. Teachers with different levels of psychological empowerment may differ in how strongly their perceptions of leadership are associated with professional development-related experiences and activities, suggesting that psychological empowerment may constitute an important boundary condition in the relationship between aesthetic leadership and teacher professional development (Spreitzer, 1995; Seibert et al., 2011; Kim and Beehr, 2023). However, few studies have incorporated both an experiential process and a psychological boundary condition into a single analytical framework. Empirical evidence concerning these processes remains particularly limited in the context of Chinese high schools (Lin et al., 2023).

Accordingly, this study focuses on high school teachers in Hainan Province, China, and examines the association between perceived principal aesthetic leadership and teacher professional development. It further tests the statistical indirect role of aesthetic experience and the moderating role of psychological empowerment. This study extends previous research in three ways. First, building on the work-autonomy pathway examined by Yin and Chang (2025), it shifts attention to aesthetic experience as a distinct experiential process associated with the relationship between perceived principal aesthetic leadership and teacher professional development. Second, it incorporates psychological empowerment as a boundary condition to examine whether the strength of the relevant associations differs across levels of teachers’ psychological resources. Third, it integrates aesthetic experience and psychological empowerment within a conditional process framework, thereby providing a more differentiated account of the experiential and psychological conditions associated with teacher professional development. Through these analyses, this study seeks to provide additional empirical evidence for educational leadership research in the context of Chinese high schools and to offer theoretical implications for understanding teacher professional development through the joint consideration of aesthetic experiences and psychological resources.

## 2 Literature review and hypotheses

Aesthetic leadership theory conceptualizes leadership not only as a process of goal management and institutional control but also as an aesthetic practice through which organizational members’ experiences may be shaped by perception, emotion, symbolism, and context (Hansen et al., 2007; Howard, 1996). In school settings, principals may foster teachers’ identification and professional engagement by cultivating the cultural atmosphere, providing emotional care, and communicating educational meaning, which may subsequently be associated with teacher professional development (Bates and Stanley, 2006; Lin et al., 2023). Meanwhile, the social interaction perspective emphasizes that teachers do not passively receive principals’ leadership behaviors or school culture. Rather, their understanding develops through the continuous interpretation and construction of meaning in everyday interactions (Blumer, 1986; Scott and Myers, 2010). Accordingly, the association between perceived principal aesthetic leadership and teacher professional development may depend not only on leadership behaviors themselves but also on the experiences and psychological responses that teachers develop through their interactions with school leaders. On this basis, aesthetic leadership theory and the social interaction perspective provide a theoretical foundation for understanding the relationships among perceived principal aesthetic leadership, aesthetic experience, psychological empowerment, and teacher professional development.

### 2.1 Perceived principals’ aesthetic leadership and teacher professional development

Teacher professional development involves not only the continuous updating of teachers’ knowledge and instructional competencies but also participation in developmental activities, such as experimenting with teaching practices, engaging in professional reflection, seeking feedback, collaborating with colleagues to improve classroom instruction, and contributing collaboratively to school improvement (Evers et al., 2016; Ingvarson et al., 2005; Sancar et al., 2021). Previous research has shown that the vision, professional support, collaborative climate, and learning opportunities provided by school leaders are closely associated with teachers’ continuous learning, instructional improvement, and professional practices (Hallinger and Heck, 2010; Liu and Hallinger, 2018). These findings suggest that teacher professional development is related not only to teachers’ individual willingness to learn but also to the everyday working environment and professional culture fostered by school leaders.

Compared with leadership approaches that primarily emphasize institutional management, goal coordination, and rational decision-making, aesthetic leadership places greater emphasis on how leaders’ language, behavior, cultural symbolism, emotional interactions, and shaping of the environment relate to organizational members’ overall experiences of school life and their interpretations of its meaning (Howard, 1996; Hansen et al., 2007). When teachers perceive principals’ communication, conduct, and cultural practices as coherent, emotionally engaging, and educationally meaningful, they may be more likely to identify with the professional values promoted by the school and to participate actively in developmental activities, including updating instructional content, experimenting with classroom practices, reflecting professionally, and collaborating with colleagues (Bathurst and Monin, 2010). Related studies have also identified significant associations between principals’ leadership behaviors and teachers’ innovative teaching, creative practices, and professional learning behaviors (Hsieh et al., 2021; Lin et al., 2023).

Accordingly, the positive association between teachers’ perceptions of principals’ aesthetic leadership and their professional development may be reflected in greater participation in activities involving instructional updating, teaching experimentation, professional reflection, feedback seeking, collaboration with colleagues to improve classroom instruction, and collective efforts to advance school development. Based on this reasoning, the following hypothesis is proposed:

*H1*: High school teachers’ perceptions of principals’ aesthetic leadership are positively associated with their professional development.

### 2.2 Perceived principals’ aesthetic leadership and aesthetic experience

Aesthetic experience refers to an integrated experience that arises when individuals encounter situations characterized by formal qualities, emotional intensity, and symbolic meaning. It primarily encompasses aesthetic pleasure, aesthetic attitudes, an understanding of beauty, and a holistic experience of the situation (Chang, 2017). Aesthetic experience is not confined to artistic creation or appreciation; it may also emerge from teachers’ everyday experiences of school spaces, organizational culture, leadership communication, and educational activities (Hobbs, 2012; Hobbs and Kelly, 2017).

Aesthetic leadership theory proposes that leadership activities involve not only formal decision-making and administrative management but also the shaping of the environment, symbolic expression, emotional communication, and leaders’ embodied presence. These aesthetic cues may be closely associated with organizational members’ perceived quality of the work environment and their level of aesthetic engagement (Hansen et al., 2007; Howard, 1996). In school settings, when teachers perceive aesthetic qualities in principals’ communication, management practices, and cultural expressions, they may be more likely to experience harmony, respect, meaningfulness, and professional enjoyment in school life. They may also develop more positive aesthetic attitudes and a more holistic experience of their school environment (Lin et al., 2023). Previous research has similarly identified significant relationships between aesthetic practices in educational environments and teachers’ aesthetic attitudes, quality of experience, and engagement in education (Lin et al., 2023; Lin et al., 2022).

In summary, teachers’ perceptions of principals’ aesthetic leadership may be specifically associated with their aesthetic pleasure, aesthetic attitudes, understanding of beauty, and holistic experience, rather than merely with generally positive feelings about work. Based on this reasoning, the following hypothesis is proposed:

*H2*: High school teachers’ perceptions of principals’ aesthetic leadership are positively associated with their aesthetic experience.

### 2.3 Aesthetic experience and teacher professional development

Aesthetic experience encompasses not only individuals’ emotional responses to a situation but also their attention to, understanding of, reflection on, and construction of meaning in educational practice (Hobbs, 2012; Hobbs and Kelly, 2017). When teachers develop more positive aesthetic experiences in their teaching and school lives, they may interpret classroom interactions, instructional forms, and student feedback with greater sensitivity. They may also be more willing to reflect on their existing teaching practices, experiment with new classroom approaches, and participate in professional exchanges (Albers et al., 2019).

Previous studies have shown that professional learning activities characterized by aesthetic and creative elements are closely associated with teachers’ reflective practices, instructional renewal, and professional engagement (Albers et al., 2019; Anderson et al., 2022). When teachers experience greater aesthetic pleasure, aesthetic understanding, and a stronger sense of wholeness in their work, they may become more sensitive to classroom interactions, instructional forms, and student feedback and more willing to reflect on established teaching practices, experiment with new classroom approaches, and engage in professional exchanges (Anderson et al., 2022; Lin et al., 2023). Aesthetic experience is therefore not merely a generally positive emotional state; it may also be regarded as an experiential psychological resource associated with professional development activities such as reflection, experimentation, instructional renewal, and collaboration. Based on this reasoning, the following hypothesis is proposed:

*H3*: High school teachers’ aesthetic experience is positively associated with their professional development.

### 2.4 The mediating role of aesthetic experience

From a social interaction perspective, the relationship between leadership behaviors and organizational members’ subsequent attitudes and behaviors does not arise automatically from the leadership behaviors themselves. Rather, it may depend on how organizational members interpret leaders’ language, conduct, emotional expressions, and symbolic practices in their everyday interactions (Blumer, 1986; Scott and Myers, 2010). Accordingly, the relationship between teachers’ perceptions of principals’ aesthetic leadership and their professional development may depend partly on how teachers experience the school life shaped by their principals.

In the context of the present study, teachers’ perceptions of principals’ aesthetic leadership may first be associated with their aesthetic experiences of school culture, the emotional atmosphere, and symbolic expressions. These aesthetic experiences may, in turn, be associated with professional development activities such as updating instructional content, experimenting with teaching practices, engaging in professional reflection, seeking feedback, and collaborating with colleagues (Hobbs and Kelly, 2017; Lin et al., 2023). When teachers perceive principals’ communication, cultural expression, environmental arrangements, and conduct as coherent, emotionally engaging, and educationally meaningful, they may experience greater aesthetic pleasure, develop more positive aesthetic attitudes, and construct a more holistic sense of meaning in school life (Chang, 2017; Hansen et al., 2007). Such aesthetic experiences may subsequently be associated with teachers’ willingness to reflect on their teaching, experiment with new classroom approaches, seek professional feedback, and collaborate with colleagues (Keung et al., 2020). Aesthetic experience may therefore be understood as an experiential link between teachers’ perceptions of external leadership practices and their participation in professional development activities.

It should be noted that this study employs a cross-sectional design. It therefore examines a statistical indirect association among the variables rather than a causal mechanism with an established temporal sequence. Based on this reasoning, the following hypothesis is proposed:

*H4*: High school teachers’ perceptions of principals’ aesthetic leadership are positively and indirectly associated with their professional development through aesthetic experience.

### 2.5 The moderating role of psychological empowerment

Psychological empowerment reflects individuals’ overall perceptions of meaning, competence, self-determination, and impact at work and represents an important psychological condition for understanding how teachers evaluate leadership support and organizational resources (Seibert et al., 2011; Spreitzer, 1995). Previous research has shown that higher levels of psychological empowerment are closely associated with more positive evaluations of work resources, greater work engagement, innovative behavior, and developmental outcomes (Kim and Beehr, 2023; Seibert et al., 2011).

In educational settings, teachers with higher levels of psychological empowerment generally report stronger perceptions of work meaning, competence, self-determination, and impact. They may therefore be more likely to interpret the aesthetic values, emotional support, and cultural care communicated by principals as organizational resources that can be applied to instructional improvement and professional learning (Ugaddan and Park, 2016). In contrast, although teachers with lower levels of psychological empowerment may recognize the aesthetic qualities of principals’ leadership behaviors, their relatively limited sense of autonomy, competence, or impact may make them less likely to connect these leadership perceptions with specific instructional improvement and professional development activities (Kim and Beehr, 2023).

From this perspective, psychological empowerment can be understood primarily as a psychological resource that supports teachers’ autonomous professional action. Teachers’ perceptions of principals’ aesthetic leadership represent an external leadership resource, whereas teacher professional development is reflected in developmental activities such as updating instructional content, experimenting with teaching practices, engaging in professional reflection, seeking feedback, and collaborating with colleagues. Teachers with higher levels of psychological empowerment may be better able to apply perceived leadership support, cultural values, and professional opportunities to these activities. Accordingly, this study first proposes that psychological empowerment changes the strength of the direct association between perceived principal aesthetic leadership and teacher professional development.

The role of psychological empowerment may differ across the other paths in the model. The association between aesthetic leadership and aesthetic experience primarily reflects how teachers perceive and experience principals’ language, cultural symbols, emotional atmosphere, and environmental arrangements. It therefore concerns perceptual and experiential evaluations of aesthetic cues. In contrast, psychological empowerment reflects teachers’ perceptions of work meaning, competence, self-determination, and impact rather than their aesthetic sensitivity or receptiveness to aesthetic cues. Thus, there is insufficient theoretical justification for predicting that psychological empowerment will systematically alter the association between aesthetic leadership and aesthetic experience.

By comparison, the association between aesthetic experience and teacher professional development concerns whether teachers connect aesthetic pleasure, meaningful experiences, and a sense of wholeness with developmental activities such as teaching experimentation, professional reflection, feedback seeking, and collaboration with colleagues. This process involves not only teachers’ experiences of school life but also their capacity to take autonomous professional action. Psychological empowerment may therefore moderate not only the direct association between aesthetic leadership and teacher professional development but also the association between aesthetic experience and teacher professional development. Consequently, the indirect association between aesthetic leadership and teacher professional development through aesthetic experience may vary across levels of psychological empowerment. However, existing theoretical and empirical evidence is insufficient to specify the direction of this conditional association in advance. Therefore, this study proposes a directional hypothesis for the direct path between aesthetic leadership and teacher professional development and examines the role of psychological empowerment in the other paths as exploratory research questions.

*H5*: Psychological empowerment moderates the positive association between high school teachers’ perceptions of principals’ aesthetic leadership and their professional development, such that this positive association is stronger among teachers with higher rather than lower levels of psychological empowerment.

*RQ1a*: Does psychological empowerment moderate the association between high school teachers’ perceptions of principals’ aesthetic leadership and their aesthetic experience?

*RQ1b*: Does psychological empowerment moderate the association between aesthetic experience and teacher professional development, such that the indirect association between aesthetic leadership and teacher professional development through aesthetic experience varies across levels of psychological empowerment?

## 3 Materials and methods

**Figure 1:**
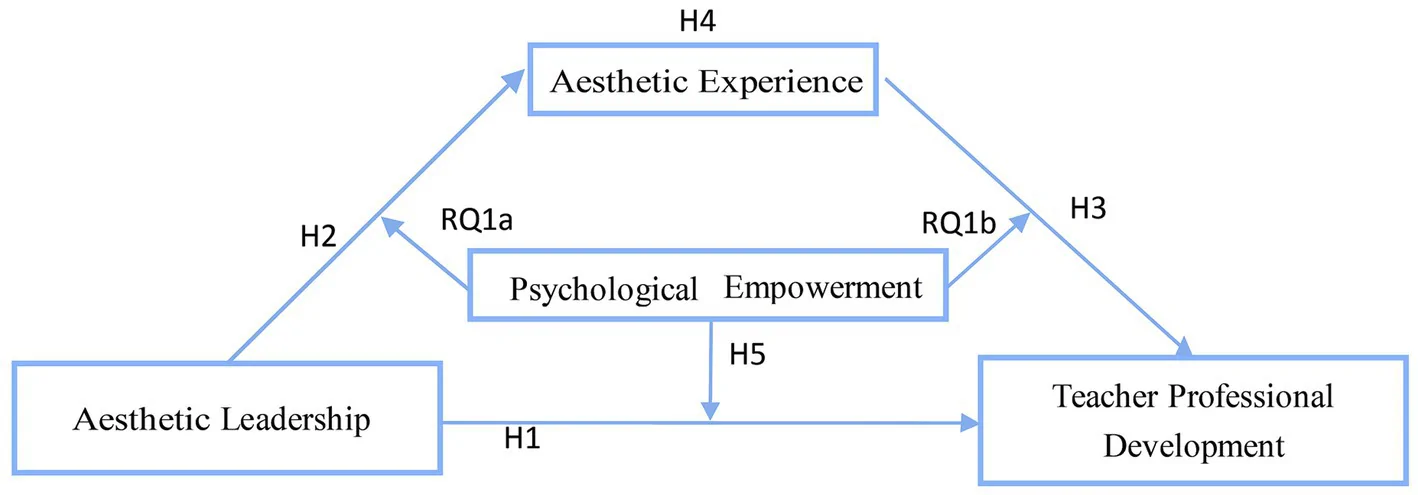
Research framework.

Drawing on aesthetic leadership theory and the social interaction perspective, this study focused on high school teachers in Hainan Province, China, and examined the relationships among perceived principal aesthetic leadership, teacher professional development, aesthetic experience, and psychological empowerment (Hansen et al., 2007; Scott and Myers, 2010). Specifically, perceived principal aesthetic leadership was treated as the independent variable, teacher professional development as the dependent variable, aesthetic experience as the mediator, and psychological empowerment as the moderator. As shown in Figure 1, the conceptual model specified the indirect association between AL and TPD through AE and further examined whether PE moderated the AL–AE, AL–TPD, and AE–TPD associations, as well as whether the indirect association between AL and TPD through AE varied across levels of PE (Hayes, 2013; Preacher et al., 2007).

### 3.1 Participants and sampling design

The target population comprised teachers employed in general high schools in Hainan Province, China. The study examined the association between teachers’ perceptions of principals’ aesthetic leadership and their professional development. Hainan Province was selected because of its ongoing educational reforms, regional cultural diversity, and variation in school development, providing a relevant context for examining aesthetic leadership in Chinese high schools (Lin et al., 2023).

The study was conducted in two stages: a pilot study and a formal survey. The pilot study assessed the appropriateness of item wording and scale structures, whereas the formal survey was used to test the hypotheses. A stratified sampling strategy was used at the school-selection stage, taking regional distribution and teachers’ age, educational attainment, and teaching experience into account (Creswell, 2014; Fowler, 2014). Participants were recruited from eight general high schools in Haikou and Sanya, including five public and three private schools. Although the sample covered diverse teacher characteristics within the selected schools, it should not be considered fully representative of all high school teachers in Hainan Province or China. All participants provided informed consent and completed the questionnaire anonymously.

The final analytical sample consisted of 611 valid questionnaires. The formal-sample dataset was also used in Yin and Chang (2025), which examined work autonomy as a mediator between perceived principals’ aesthetic leadership and teacher professional development. The present secondary analysis addresses a different research question, focusing on aesthetic experience as a statistical intermediary and psychological empowerment as a boundary condition; the earlier work-autonomy mediation model is not re-tested here. Of the participants, 221 were men (36.17%) and 390 were women (63.83%). Teachers aged 35–49 years constituted the largest age group (43.21%), followed by those aged 25–34 years (37.48%). Most participants held a bachelor’s degree or lower qualification (63.34%), while 30.28% held a master’s degree and 6.38% held a doctoral degree. The largest teaching-experience groups were 5–10 years (40.92%) and 11–20 years (24.88%).

### 3.2 Instruments

The questionnaire comprised four core scales measuring perceived principal aesthetic leadership (AL), teacher professional development (TPD), aesthetic experience (AE), and psychological empowerment (PE). All four scales used a five-point Likert response format, with higher scores indicating higher perceived levels of the corresponding constructs. The use of a consistent response format enhanced comparability across the constructs and facilitated the subsequent reliability, validity, and path analyses (DeVellis, 2017; Field, 2013). The four scales contained a total of 99 items. Accordingly, the target sample size also took into account the commonly applied guideline of obtaining a sample three to five times the total number of items to support the stability of the subsequent structural equation modeling analyses.

#### 3.2.1 Scale of perceived principals’ aesthetic leadership

Perceived principals’ aesthetic leadership was assessed using an adapted version of the 51-item, seven-dimension Aesthetic Leadership Scale developed by Polat and Öztoprak-Kavak (2011). Following expert review and item analysis for the Chinese high-school context, nine items were removed, resulting in a final 42-item version that retained the original seven-dimensional structure (see Supplementary Table S2 for details). In the present study, the Cronbach’s alpha coefficient for the formal survey was .955.

#### 3.2.2 Teacher professional development scale

Teacher professional development was assessed using an adapted version based on the initial 25-item pool reported by Evers et al. (2016). The items were adapted to the Chinese high-school context. Following pilot testing and item analysis, one item from the CM1 dimension was removed because of its unsatisfactory item performance, resulting in a final 24-item version used in the formal survey. The adapted measure comprised five dimensions: continuous updating of teaching content, instructional experimentation, reflection and feedback seeking, collaboration with colleagues to improve classroom teaching, and collaboration with colleagues to promote school development. In the present study, the items were rated on a five-point frequency scale ranging from 1 (hardly ever) to 5 (always). The scale demonstrated excellent internal consistency (Cronbach’s *α* = .947).

#### 3.2.3 Aesthetic experience scale

Aesthetic experience was measured using the aesthetic experience scale developed by Chang (2017). The scale contains 21 items across four dimensions: pleasure derived from beauty, aesthetic attitudes, understanding of beauty, and holistic experience. Lin et al. (2022) demonstrated the applicability of this scale to teachers in a study of university faculty members in China. In the formal survey, the scale demonstrated excellent internal consistency, with a Cronbach’s alpha of 0.924.

#### 3.2.4 Psychological empowerment scale

Psychological empowerment was measured using the 12-item scale developed by Spreitzer (1995). The scale comprises four dimensions: meaning, competence, self-determination, and impact. Li et al. (2006) translated and back-translated the scale, conducted questionnaire-based interviews, and validated the reliability and construct validity of its four-dimensional structure in the Chinese context. In the formal survey, the scale demonstrated good internal consistency, with a Cronbach’s alpha of 0.873.

### 3.3 Pilot testing, scale revision, and formal survey

To improve the contextual appropriateness of the questionnaire, the scales underwent translation and adaptation, expert review, and pilot testing before the formal survey was conducted (Beaton et al., 2000; Boateng et al., 2018). Scales originally developed in English were first translated into Chinese, and selected expressions were adapted to the context of high school teachers in Hainan Province, China, to improve semantic clarity and cultural relevance. Each English-language scale was independently translated into Chinese by two bilingual experts familiar with educational research. After the two translations had been compared and reconciled, two additional experts who had not participated in the initial translation independently translated the Chinese version back into English. The research team then compared the original and back-translated versions and revised the Chinese wording until conceptual equivalence was achieved. Throughout this process, priority was given to preserving the semantic meaning of the original items, while culturally specific differences were addressed where necessary.

After the translation process, eight experts in education and psychology were invited to evaluate the content validity of the scale items. Each item was rated on a four-point Likert scale ranging from 1 (not relevant) to 4 (highly relevant), and the content validity index (CVI) was calculated. The CVI for each scale exceeded 0.80, indicating satisfactory content validity. Item wording was revised where modifications were recommended by the experts. (e.g., Supplementary Tables S1 and S2).

A total of 301 valid questionnaires were obtained in the pilot study. Based on the pilot data, item analysis and reliability testing were conducted, and several items were screened and revised accordingly. The formal survey strictly followed research ethics requirements. All participants completed the questionnaire anonymously on the basis of informed consent. The data collection procedure was approved by the ethics committee of Dhurakij Pundit University (DPU BSH 1607/2567). Questionnaires were completed anonymously to reduce evaluation apprehension and social desirability concerns; however, common-method variance could not be ruled out because all focal variables were self-reported in a single survey wave (Podsakoff et al., 2003).

### 3.4 Data analysis

Because the participants were recruited from different schools, clustering effects for the core variables were assessed before the main analyses. ICC (1), ICC (2), and the design effect were calculated to evaluate the potential influence of school-level nesting on the parameter estimates.

The data analysis was conducted in two stages: measurement model assessment and conditional process analysis. In the first stage, confirmatory factor analyses were performed in AMOS to assess the measurement structures of AL, AE, TPD, and PE, including measurement quality, convergent validity, and discriminant validity. A latent-variable structural equation model was subsequently used to examine the direct association between AL and TPD and the statistical indirect association between AL and TPD through AE. By accounting for measurement error, this latent-variable mediation analysis was used to test H1–H4 and to provide robustness evidence for the mediation results.

In the second stage, SPSS PROCESS Model 59 was used to test the complete conceptual model within a unified observed-variable conditional process framework. Composite scores for the AL, AE, TPD, and PE scales were used, and gender, age, educational attainment, and teaching experience were included as covariates. The AL × PE interaction term was entered into the regression equation predicting AE to examine whether PE moderated the association between AL and AE. The AL × PE and AE × PE interaction terms were simultaneously entered into the regression equation predicting TPD to examine whether PE moderated the direct association between AL and TPD and the association between AE and TPD, respectively. The model also estimated the conditional indirect association between AL and TPD through AE, thereby testing H5 and the exploratory research questions RQ1a and RQ1b.

Before the interaction terms were constructed, AL, AE, and PE were mean-centered. Conditional direct and indirect effects were estimated at low, mean, and high levels of PE, with the low and high levels defined as one standard deviation below and above the mean, respectively. 95% confidence intervals were calculated using 5,000 bootstrap resamples. When an interaction term was statistically significant, its incremental explained variance (Δ*r*^2^) was also reported to evaluate both statistical significance and its additional explanatory contribution. A conditional indirect association was considered statistically significant when its bootstrap confidence interval did not include zero. Regression assumptions were assessed separately for the equations predicting AE and TPD. Residual normality was evaluated using normal Q–Q plots, whereas linearity and homoscedasticity were examined using residual-versus-fitted plots and the Breusch–Pagan and White tests. Because heteroscedasticity was detected in both equations, HC3 heteroscedasticity-consistent standard errors were used to evaluate the robustness of the regression estimates. Models containing quadratic terms for the continuous predictors were also estimated as a sensitivity analysis to assess possible functional-form departures.

AMOS and PROCESS served distinct rather than competing analytical purposes in this study. AMOS was used primarily to assess the measurement models and to evaluate the robustness of the mediation results after accounting for measurement error. PROCESS Model 59 was used to estimate the direct associations, indirect association, interaction terms, and conditional indirect effects simultaneously within a single observed-variable framework. Accordingly, the latent-variable path coefficients obtained from AMOS were not numerically compared with the observed-variable regression coefficients obtained from PROCESS. Interpretation of the conditional process results for the complete conceptual model was based primarily on the unified PROCESS analysis. It should be noted that conditional process analysis based on composite scale scores cannot account for measurement error as fully as a latent-variable interaction model. This limitation is discussed further in the limitations section (Hayes, 2013; Preacher et al., 2007).

## 4 Results

### 4.1 Common method bias test

Because all focal variables were obtained from the same respondents using self-report measures within a single survey wave, common-method variance cannot be ruled out. Harman’s single-factor analysis showed that the first factor accounted for 33.32% of the total variance, indicating that no single factor dominated the unrotated factor solution. However, Harman’s procedure is only a limited diagnostic and does not establish the absence of common-method bias. Accordingly, common-method variance remains a potential limitation, and the observed associations should be interpreted cautiously.

### 4.2 Results of the clustering effect test

Given that the sample was drawn from eight high schools, clustering effects were examined before the main analyses. The results showed that the ICC (1) values for principals’ aesthetic leadership, teacher professional development, and aesthetic experience were approximately zero, indicating negligible between-school variance. For psychological empowerment, the ICC (1) was 0.009 and the design effect was 1.698, which remained below the commonly used threshold of 2.0. Overall, the clustering effects were weak, suggesting that subsequent analyses at the individual teacher level were appropriate (Table 1).

**Table 1:** Results of the clustering effect test (*n* = 611).

Variable	ICC (1)	ICC (2)	Design effect
AL	0.000	–	1.000
TPD	0.000	–	1.000
AE	0.000	–	1.000
PE	0.009	0.416	1.698

AL, perceived principals’ aesthetic leadership; AE, aesthetic experience; TPD, teacher professional development; PE, psychological empowerment.

### 4.3 Correlation analysis

Pearson product–moment correlations were calculated to examine the linear relationships among perceived principal aesthetic leadership, teacher professional development, aesthetic experience, and psychological empowerment, providing a basis for the subsequent regression and conditional process analyses (Schober et al., 2018). The results are presented in Table 2.

**Table 2:** Descriptive statistics and correlations among the four variables (*n* = 611).

Variable	Mean	SD	AL	TPD	AE	PE
AL	3.502	0.771	1			
TPD	3.604	0.838	0.510^***^	1		
AE	3.766	0.785	0.501^***^	0.536^***^	1	
PE	2.664	0.714	0.451^***^	0.457^***^	0.424^***^	1

^***^p < 0.001. AL, perceived principals’ aesthetic leadership; TPD, teacher professional development; AE, aesthetic experience; PE, psychological empowerment.

### 4.4 Structural equation model fit test

In the AMOS structural model, perceived principal aesthetic leadership (AL), aesthetic experience (AE), and teacher professional development (TPD) were specified as latent variables, with their seven, four, and five dimension-level composite scores, respectively, used as observed indicators. The use of dimension-level indicators preserved the multidimensional theoretical structures and construct coverage of the original scales. Moreover, the three scales contained a total of 87 items, and including all items simultaneously in the structural model would have substantially increased model complexity and the number of parameters to be estimated. The use of dimension-level composite indicators therefore improved model parsimony and estimation stability. Before the structural model was estimated, item-level confirmatory factor analyses were conducted separately for each scale. The standardized factor loadings of the items, composite reliability values, and average variance extracted values for the dimensions are reported in Supplementary Tables S3–S6.

Because AMOS was primarily used to examine the structural relationships in the mediation model, the fit of the three-variable model comprising AL, AE, and TPD was assessed first. The model-fit indices showed a mixed but generally acceptable pattern. χ^2^/df = 1.748, RMSEA = 0.035, CFI = 0.940, IFI = 0.940, and TLI = 0.938 were broadly supportive of acceptable model fit. However, GFI = 0.803 was relatively modest and NFI = 0.870 was lower than the commonly used 0.900 benchmark. Therefore, the model should not be characterized as exhibiting uniformly strong fit (Harrington, 2009). Taken together, the indices suggest an acceptable overall approximation to the observed data, while some indices indicate room for improvement, shown in Table 3.

**Table 3:** Summary of structural model fit indices (*n* = 611).

Model fit index	Criterion	Observed value
χ2/df	<5	1.748
GFI	>0.800	0.803
RMSEA	<0.080	0.035
CFI	>0.900	0.940
NFI	>0.900	0.870
IFI	>0.800	0.940
TLI	>0.800	0.938
PGFI	>0.500	0.762
PNFI	>0.500	0.845
PCFI	>0.500	0.913

**Figure 2:**
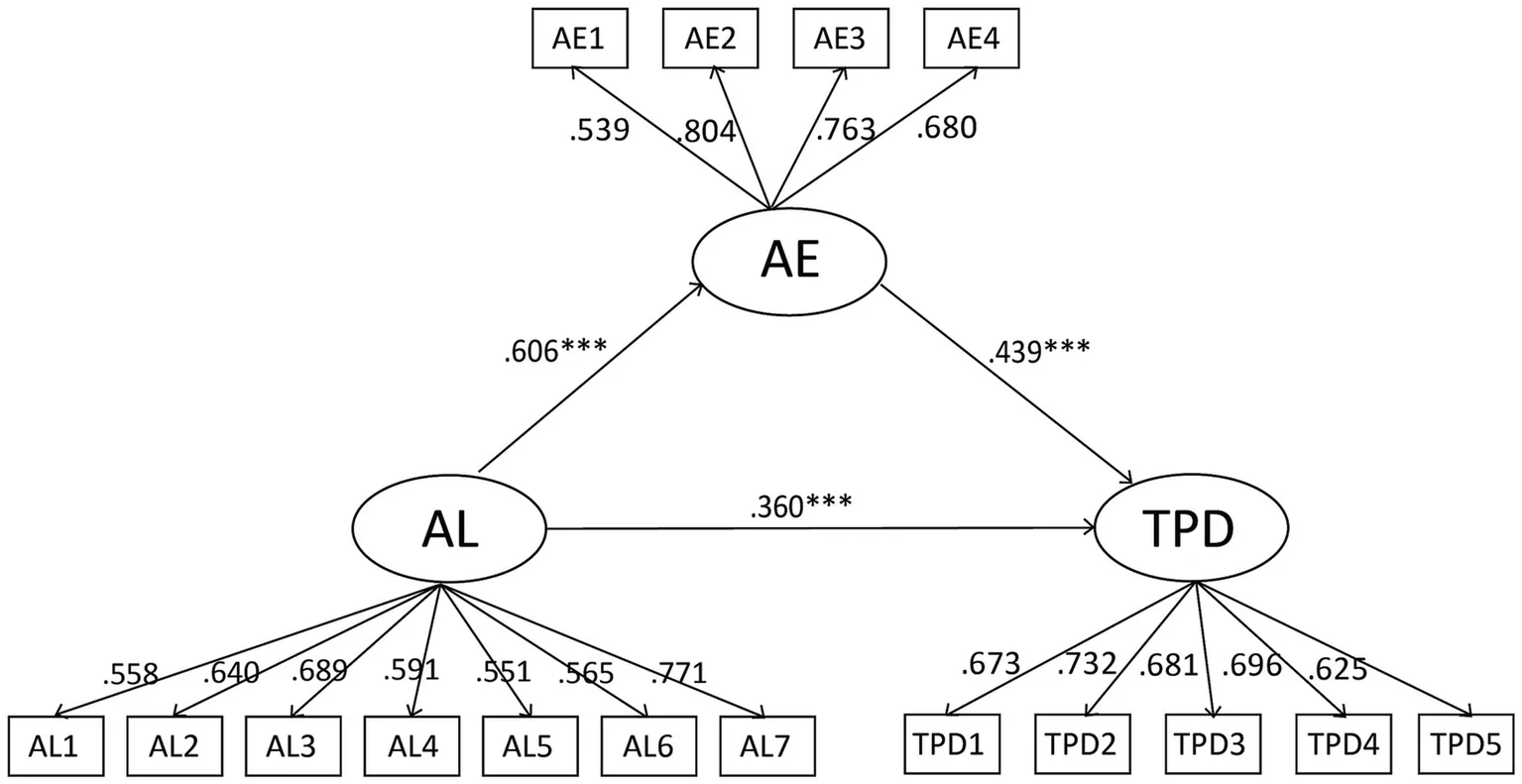
Mediation model. *p < 0.05, **p < 0.01, ***p < 0.001. AL = perceived principals’ aesthetic leadership; AE = aesthetic experience; TPD = teacher professional development.

With the structural model demonstrating an acceptable overall fit, the mediating role of aesthetic experience in the relationship between perceived principals’ aesthetic leadership and teacher professional development was further tested. The mediation model is shown in Figure 2.

### 4.5 Latent-variable mediation analysis

To determine whether the statistical indirect association between AL and TPD through AE remained robust after accounting for measurement error, a latent-variable mediation analysis was conducted in AMOS. This analysis was used primarily to test H1–H4 and to provide robustness evidence for the mediation findings. The moderating effects and conditional indirect effects in the complete conceptual model were subsequently examined using the unified PROCESS conditional process model.

As shown in Table 4, the indirect role of AE was further tested in AMOS using 5,000 bootstrap resamples. The direct association between AL and TPD was statistically significant (effect = 0.360, 95% CI [0.208, 0.497]), supporting H1. The path from AL to AE was also statistically significant (effect = 0.606, 95% CI [0.511, 0.691]), supporting H2, and the path from AE to TPD was statistically significant (effect = 0.439, 95% CI [0.297, 0.592]), supporting H3. In addition, the indirect effect of AL on TPD through AE was 0.266, with a 95% confidence interval of [0.175, 0.385]. Because the confidence interval did not include zero, the statistical indirect association was significant, supporting H4. The direct path between AL and TPD remained statistically significant after AE was included in the model, indicating partial statistical mediation. These latent-variable findings provided robustness evidence for the proposed mediation relationship.

**Table 4:** Bootstrap analysis of the mediating effect of aesthetic experience (*n* = 611).

Effect type	Path	Effect value	95% CI (LLCI)	95% CI (ULCI)
Direct effect	AL → TPD	0.360^***^	0.208	0.497
X → M	AL → AE	0.606^***^	0.511	0.691
M → Y	AE → TPD	0.439^***^	0.297	0.592
Indirect effect	AL → AE → TPD	0.266^***^	0.175	0.385
Total effect	AL → TPD	0.627^***^	0.538	0.707

AL, perceived principals’ aesthetic leadership; AE, aesthetic experience; TPD, teacher professional development; The table reports the effect estimates and their 95% confidence intervals obtained from 5,000 Bootstrap resamples; LLCI, lower limit of the confidence interval; ULCI, upper limit of the confidence interval.

### 4.6 Test of the moderating effect of psychological empowerment

Following the latent-variable mediation analysis conducted in AMOS, PROCESS Model 59 was used to examine the complete model within a unified observed-variable conditional process framework. Specifically, the analysis simultaneously tested whether PE moderated the association between AL and AE, the direct association between AL and TPD, and the association between AE and TPD. It also estimated the conditional indirect association between AL and TPD through AE at different levels of PE. Gender, age, educational attainment, and teaching experience were included as covariates, and all continuous variables were mean-centered before the interaction terms were constructed. Table 5 reports standardized regression coefficients, whereas the conditional effects reported in Table 6 are unstandardized estimates.

**Table 5:** Conditional process analysis of psychological empowerment in the associations among perceived principal aesthetic leadership, aesthetic experience, and teacher professional development (*n* = 611).

Variable	Model 1			Model 2			Model 3		
	*β*	*t*	VIF	*β*	*t*	VIF	*β*	*t*	VIF
Female	0.076^*^	2.227	1.010	0.020	0.637	1.018	0.019	0.618	1.019
25–34 years	0.008	0.127	3.787	0.064	1.050	3.776	0.079	1.318	3.790
35–49 years	0.073	1.093	3.870	0.042	0.679	3.870	0.054	0.889	3.879
50 years and above	−0.005	−0.095	2.327	−0.026	−0.538	2.319	−0.004	−0.082	2.341
Master’s degree	−0.040	−1.133	1.070	−0.088^**^	−2.714	1.072	−0.085^**^	−2.645	1.074
Doctoral degree	−0.021	−0.592	1.058	−0.013	−0.401	1.059	−0.005	−0.147	1.062
5–10 years	−0.081	−1.740	1.912	0.002	0.036	1.920	0.002	0.039	1.923
11–20 years	−0.092^*^	−2.033	1.786	0.013	0.315	1.799	0.016	0.378	1.799
More than 20 years	−0.044	−1.029	1.590	−0.000	−0.005	1.593	0.004	0.098	1.595
AL	0.381^***^	9.817	1.323	0.247^***^	6.414	1.498	0.238^***^	6.214	1.540
AE	–	–	–	0.312^***^	8.188	1.468	0.280^***^	7.328	1.535
Moderator									
PE	0.248^***^	6.460	1.294	0.211^***^	5.754	1.365	0.233^***^	6.396	1.392
Interaction terms									
AL × PE	−0.004	−0.106	1.040	–	–	–	0.164^***^	4.345	1.485
AE × PE	–	–	–	–	–	–	−0.149^***^	−3.932	1.497
*r^2^*	0.319			0.410			0.431		
*Δr^2^*	<0.001			0.066			0.021		
*f* value	23.311^***^			34.622^***^			32.279^***^		

^*^p < 0.050, ^**^p < 0.010, ^***^p < 0.001; The reference groups were men, teachers younger than 25 years, teachers with a bachelor’s degree or lower qualification, and teachers with less than 5 years of teaching experience; AL, perceived principal aesthetic leadership; AE, aesthetic experience; TPD, teacher professional development; PE, psychological empowerment; VIF, variance inflation factor.

**Table 6:** Conditional effects at different levels of psychological empowerment (*n* = 611).

Path	Psychological empowerment	Effect	*SE*	LLCI	ULCI
AL → TPD conditional direct effect	Low PE (−0.714)	0.102	0.058	−0.012	0.216
	Mean PE (0)	0.259	0.042	0.177	0.340
	High PE (0.714)	0.415	0.052	0.313	0.518
AE → TPD conditional effect	Low PE (−0.714)	0.446	0.049	0.350	0.543
	Mean PE (0)	0.299	0.041	0.219	0.379
	High PE (0.714)	0.152	0.061	0.032	0.272
AL → AE → TPD conditional indirect effect	Low PE (−0.714)	0.175	0.038	0.105	0.254
	Mean PE (0)	0.116	0.024	0.072	0.165
	High PE (0.714)	0.058	0.030	0.004	0.119

Low and high PE represent one standard deviation below and above the mean of PE, respectively; The confidence intervals for the conditional AL → TPD and AE → TPD effects are conventional regression-based 95% confidence intervals. The conditional indirect AL → AE → TPD effects were estimated using 5,000 bootstrap resamples; LLCI and ULCI represent the lower and upper limits of the 95% confidence interval, respectively; The effect estimates reported in the table are unstandardized coefficients; AL, perceived principal aesthetic leadership; AE, aesthetic experience; TPD, teacher professional development; PE, psychological empowerment.

Residual diagnostics indicated mild departures from normality and significant heteroscedasticity in both regression equations. The Breusch–Pagan tests were significant for the equation predicting AE (*f* = 2.998, *p* < 0.001) and the equation predicting TPD (*f* = 3.908, *p* < 0.001), and the White tests produced the same conclusion. Accordingly, the models were re-estimated using HC3 heteroscedasticity-consistent standard errors. Under HC3 estimation, the AL × PE interaction remained nonsignificant in the AE equation (*b* = −0.005, SE = 0.054, *p* = 0.933). In the TPD equation, the AL × PE interaction remained significantly positive (*b* = 0.219, SE = 0.068, *p* = 0.001), whereas the AE × PE interaction remained significantly negative (*b* = −0.206, SE = 0.069, *p* = 0.003). The quadratic-term sensitivity analysis indicated some curvature, but the main interaction conclusions remained unchanged. The diagnostic plots are presented in Supplementary Figures S1–S4.

**Figure 3:**
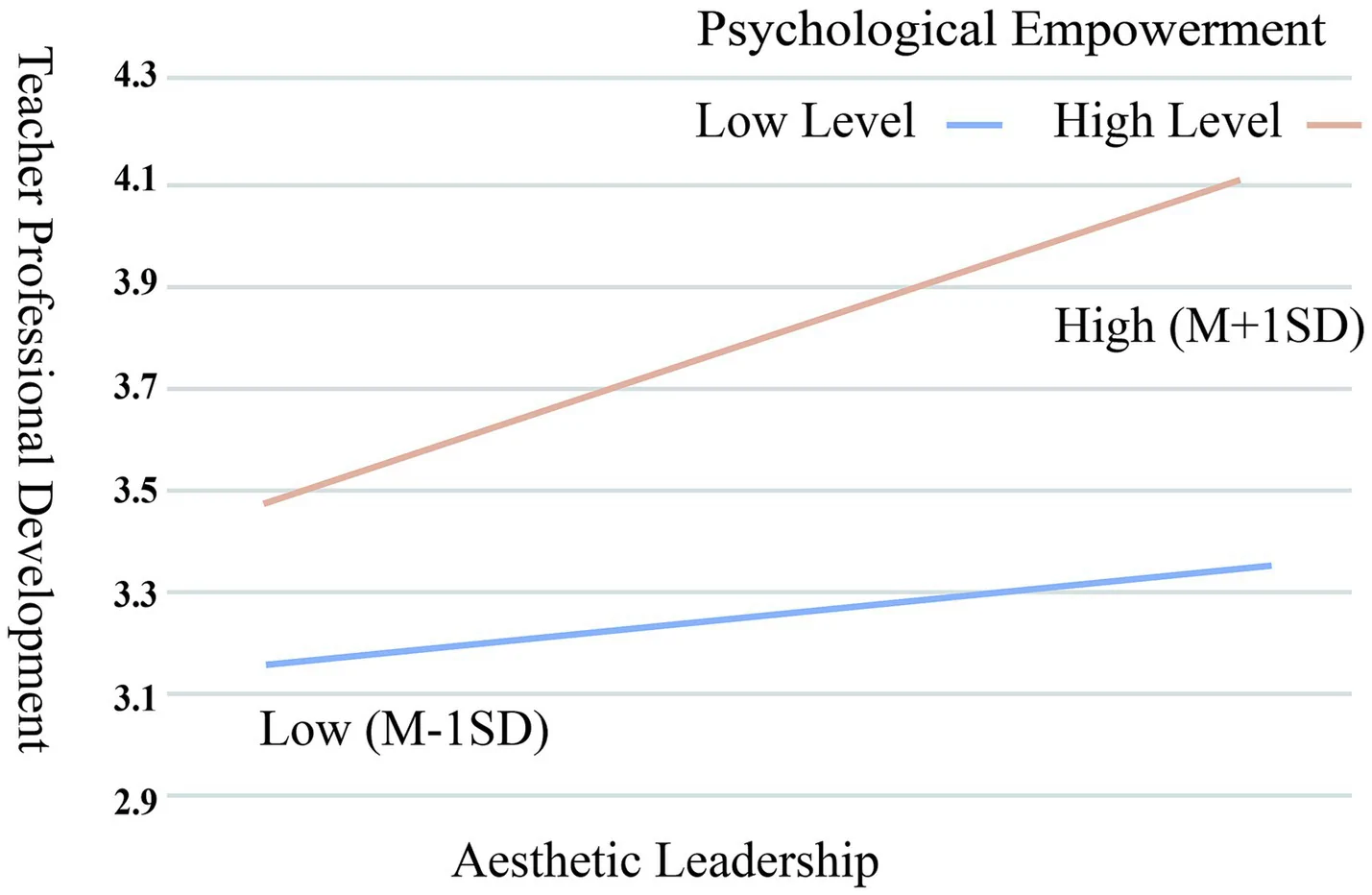
Moderating effect of psychological empowerment on the relationship between AL and TPD.

As shown in Table 5, in the regression equation predicting AE, AL was significantly and positively associated with AE (*β* = 0.381, *t* = 9.817, *p* < 0.001), and PE was also significantly and positively associated with AE (*β* = 0.248, *t* = 6.460, *p* < 0.001). However, the AL × PE interaction was not statistically significant (*β* = −0.004, *t* = −0.106, *p* = 0.916), and its incremental explained variance was less than 0.001. Thus, no statistical evidence was found that PE moderated the association between AL and AE; the strength of this association did not differ significantly across levels of PE (Figure 3).

In the regression equation predicting TPD, AL (*β* = 0.238, *t* = 6.214, *p* < 0.001), AE (*β* = 0.280, *t* = 7.328, *p* < 0.001), and PE (*β* = 0.233, *t* = 6.396, *p* < 0.001) were all significantly and positively associated with TPD. The AL × PE interaction was significantly positive (*β* = 0.164, *t* = 4.345, *p* < 0.001), whereas the AE × PE interaction was significantly negative (*β* = −0.149, *t* = −3.932, *p* < 0.001). Together, the two interaction terms increased the explained variance from *r*^2^ = 0.410 to *r*^2^ = 0.431, representing an increment of Δ*r*^2^ = 0.021. These findings indicate that the conditional role of PE was path-specific rather than uniformly strengthening all associations in the model.

**Figure 4:**
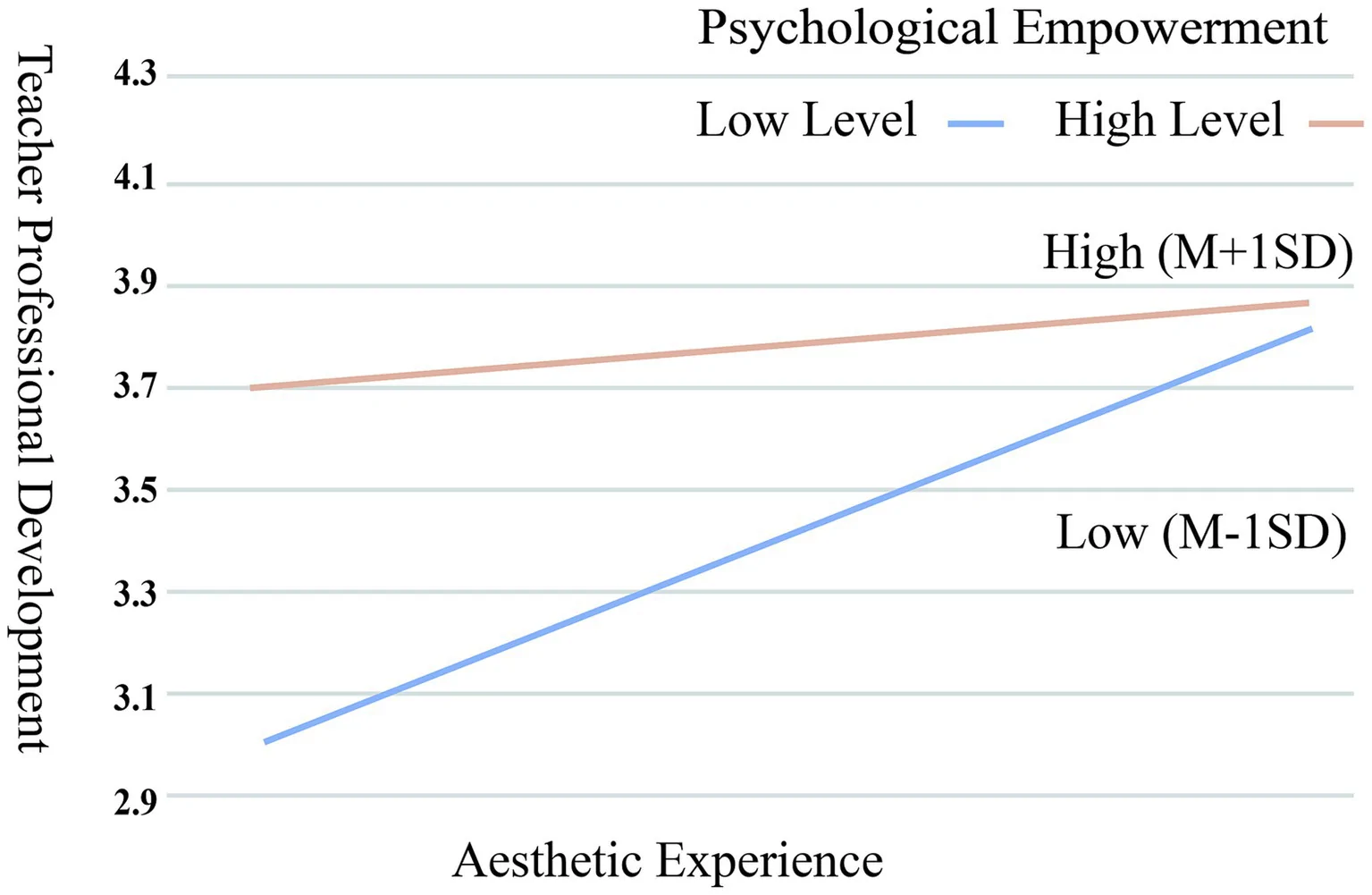
Moderating effect of psychological empowerment on the relationship between AE and TPD.

As shown in Table 6, the conditional direct effect of AL on TPD was 0.102 at a low level of PE (*SE* = 0.058, 95% CI [−0.012, 0.216]), 0.259 at the mean level of PE (*SE* = 0.042, 95% CI [0.177, 0.340]), and 0.415 at a high level of PE (*SE* = 0.052, 95% CI [0.313, 0.518]). The conditional effect of AE on TPD was 0.446, 0.299, and 0.152 at low, mean, and high levels of PE, respectively, and all three confidence intervals excluded zero. The conditional indirect effect of AL on TPD through AE was 0.175, 0.116, and 0.058 at the three corresponding levels of PE, and all bootstrap confidence intervals excluded zero. Overall, PE did not moderate the association between AL and AE, but it significantly moderated the associations from AL to TPD and from AE to TPD, such that the conditional indirect association between AL and TPD through AE weakened as PE increased (Figure 4).

## 5 Discussion

### 5.1 Perceived principal aesthetic leadership and teacher professional development

This study found a significant positive association between perceived principal aesthetic leadership (AL) and teacher professional development (TPD) among high school teachers. Teachers who perceived stronger aesthetic characteristics in principals’ communication, emotional support, cultural development, and aesthetic expression also tended to report higher levels of professional development. This finding is consistent with earlier evidence reported by Yin and Chang (2025) on the positive association between perceived principal AL and TPD. The present study builds on this earlier evidence by examining aesthetic experience and psychological empowerment as additional conditions associated with the AL–TPD relationship. This perspective suggests that teacher development may be associated not only with institutional arrangements and task management but also with teachers’ perceptions of the aesthetic qualities of school environments, communication, relationships, and organizational culture (Hansen et al., 2007; Bathurst and Monin, 2010).

From a theoretical perspective, AL emphasizes organizational members’ sensory, symbolic, and emotional experiences of leadership. Such experiences may be associated with teachers’ identification with school culture and professional behaviors involving continuous learning, instructional improvement, and collaboration (Howard, 1996; Hansen et al., 2007; Bates and Stanley, 2006). In everyday interactions, teachers may perceive respect, support, harmony, and educational meaning in principals’ leadership practices, which may in turn be associated with stronger professional identification and willingness to pursue continued growth (Scott and Myers, 2010). Taken together, the findings are consistent with the view that perceived principal AL may constitute an organizational resource associated with teacher professional development.

### 5.2 Perceived principal aesthetic leadership and aesthetic experience

This study found a significant positive association between perceived principal AL and teachers’ aesthetic experience (AE). This finding indicates that teachers who perceived stronger aesthetic characteristics in principals’ leadership behaviors were more likely to report positive aesthetic experiences in school organizational life (Hansen et al., 2007; Hobbs and Kelly, 2017). This result is consistent with the fundamental proposition of aesthetic leadership theory that leaders are associated with organizational members’ experiences not only through decision-making and management but also through emotional resonance, symbolic expression, spatial atmosphere, and organizational style, all of which may shape how members perceive and interpret the aesthetic qualities of a situation (Howard, 1996; Ropo and Sauer, 2008). In school organizations, when principals demonstrate a strong aesthetic orientation through verbal communication, behavioral modeling, environmental design, and cultural activities, teachers may be more likely to experience pleasure, harmony, meaningfulness, and respect in their everyday work. These experiences constitute important foundations of AE (Lin et al., 2023).

This finding further suggests that AE is not determined entirely by individual taste or artistic preferences but is closely associated with the organizational atmosphere and interactional environment shaped by school leadership (Hobbs, 2012; Hobbs and Kelly, 2017). Principals’ AL may be associated with a more perceptually engaging and emotionally resonant working environment in which teachers are more likely to develop stable and positive aesthetic experiences through school culture, collegial relationships, and instructional activities (Bates and Stanley, 2006; Lin et al., 2023). Accordingly, the findings are consistent with the proposition that principals’ AL is linked to teachers’ aesthetic experiences through the school organizational atmosphere and everyday interaction processes. They also provide an empirical basis for the subsequent examination of the mediating relationship (Scott and Myers, 2010).

### 5.3 Aesthetic experience and teacher professional development

This study found a significant positive association between AE and TPD. This finding indicates that teachers who reported greater aesthetic pleasure, meaningful experiences, and holistic emotional engagement at work also tended to report higher levels of professional development (Hobbs and Kelly, 2017; Anderson et al., 2022). This result is consistent with previous research linking aesthetic experience to teachers’ reflective capacity, creative practice, and occupational wellbeing. It further suggests that TPD involves not only the accumulation of knowledge and skills but also the continuing development of emotion, meaning, and experience (Albers et al., 2019). When teachers experience the “beauty of education,” the “beauty of relationships,” and the “beauty of growth” in school and classroom settings, they may be more likely to maintain high levels of instructional engagement, professional identification, and willingness to pursue continuous improvement. These characteristics are closely associated with positive manifestations of professional development (Hobbs, 2012; Lin et al., 2023).

Research on TPD has traditionally emphasized external support conditions, including training, institutional support, and professional learning communities. The present findings suggest that teachers’ aesthetic experiences of educational settings may also constitute an important internal psychological resource associated with professional growth (Avalos, 2011; Keung et al., 2020). Accordingly, explanations of why teachers continue to reflect, learn, and improve their teaching should not focus exclusively on institutional requirements or competency training. Attention should also be given to the emotional resonance, meaning-making, and aesthetic experiences that teachers develop through their work (Anderson et al., 2022; Lin et al., 2023).

### 5.4 The mediating role of aesthetic experience

This study further found that AE partially mediated the statistical association between perceived principal AL and TPD. Perceived principal AL was associated with TPD both directly and indirectly through teachers’ AE (Preacher and Hayes, 2008; Hayes, 2013). Whereas Yin and Chang (2025) identified work autonomy as an indirect pathway, the present findings indicate an additional statistical indirect association through aesthetic experience. This pattern suggests that the AL–TPD association may also involve teachers’ aesthetic, emotional, and meaning-related interpretations of principals’ leadership behaviors, although the cross-sectional design does not establish a temporally ordered causal mediation process in which teachers develop aesthetic, emotional, and meaning-related interpretations of principals’ leadership behaviors, and these experiences are subsequently associated with their motivation for professional growth (Scott and Myers, 2010; Hobbs and Kelly, 2017).

From this perspective, AE may serve as a key bridge between external leadership practices and teachers’ internal experiences of professional growth (Lin et al., 2023). Previous research on school leadership and TPD has frequently emphasized variables such as teacher efficacy, organizational commitment, work engagement, and professional identity. The present study extends this literature by suggesting that the association between leadership behaviors and teacher growth may also depend on whether teachers develop positive aesthetic experiences in organizational life (Liu and Hallinger, 2018; Anderson et al., 2022). AE is therefore not merely an individual aesthetic response; it may also represent a psychological and experiential process linking leadership practices with TPD. Its role in the model highlights the distinctive contribution of aesthetic leadership research relative to more conventional approaches to leadership (Hansen et al., 2007; Hobbs and Kelly, 2017).

The mediation identified in this study was partial rather than complete, indicating that AE represents an important, but not exclusive, indirect pathway (Hayes, 2013). Future research could therefore examine additional mediators, such as teacher engagement, professional identity, creative self-efficacy, and perceived organizational support, to develop a more comprehensive explanatory framework for the association between principal AL and TPD (Noonan, 2019).

### 5.5 The moderating role of psychological empowerment

This study found a significant interaction between psychological empowerment (PE) and AL in relation to TPD. Specifically, at a low level of PE, the conditional direct association between AL and TPD was not statistically significant. At the mean and high levels of PE, however, this conditional direct association was significantly positive and became stronger as PE increased. These findings indicate that, after controlling for AE, the positive association between teachers’ perceptions of principal AL and their TPD was relatively stronger at higher levels of PE. H5 was therefore supported. It should be emphasized that this result indicates differences in the strength of the association across levels of PE; it does not establish a causal process through which PE enables perceived leadership resources to translate into teacher professional development.

From a theoretical perspective, PE reflects teachers’ overall perceptions of work meaning, competence, self-determination, and impact. Higher levels of PE may indicate that teachers possess greater subjective resources for action. Consequently, positive perceptions of the aesthetic values, emotional support, and cultural care demonstrated by principals may be more likely to coexist with developmental activities such as teaching experimentation, professional reflection, feedback seeking, and collaboration with colleagues (Seibert et al., 2011; Spreitzer, 1995). Nevertheless, this interpretation remains a theoretically informed possibility. The current cross-sectional data cannot establish whether teachers with higher PE are more effective in utilizing leadership resources.

The exploratory results from Model 59 further indicated that the conditional role of PE was path-specific. The AL × PE interaction in predicting AE was not statistically significant, and its incremental explanatory contribution was negligible. Thus, the present study found no statistical evidence that PE altered the association between AL and AE. One possible explanation is that the AL–AE association primarily concerns teachers’ perceptual experiences of leaders’ language, cultural symbols, environmental atmosphere, and emotional interactions, whereas PE mainly reflects teachers’ judgments of their own competence, autonomy, and influence at work and is not equivalent to aesthetic sensitivity.

By contrast, the AE × PE interaction in predicting TPD was significantly negative. AE was positively associated with TPD at low, mean, and high levels of PE, but the strength of this association decreased as PE increased. Correspondingly, the statistical indirect association between AL and TPD through AE also became weaker at higher levels of PE. This pattern indicates that PE did not uniformly strengthen all associations in the model. Higher PE was associated with a stronger direct AL–TPD association but weaker AE–TPD and statistical indirect associations.

One possible explanation is that teachers with higher levels of PE may rely less on positive experiential evaluations because their professional development activities are more closely associated with their own autonomy, competence, and perceived capacity to exert influence. Teachers with lower levels of PE, by comparison, may rely more heavily on the emotional resonance, meaningful experiences, and aesthetic pleasure derived from school life, resulting in a relatively stronger association between AE and TPD. However, alternative explanations are also possible. Teachers who participate more actively in professional development may subsequently develop higher levels of PE and evaluate principals’ leadership more positively. Moreover, because all core variables were measured through self-reports from the same teachers, a general tendency toward positive evaluation may have simultaneously increased the AL, AE, PE, and TPD scores. Therefore, the current findings should not be interpreted as demonstrating a definite process in which teachers shift from “relying on aesthetic experience” to “directly utilizing leadership resources”.

### 5.6 Theoretical and practical implications

Because the present study was based on cross-sectional associations, its findings cannot demonstrate that specific principal behaviors necessarily improve teacher professional development. Nevertheless, they may inform the development of school management practices that can be evaluated in future research. Schools could establish more stable, transparent, and equitable leadership communication procedures. Before implementing curriculum reforms, teacher evaluations, or resource allocation decisions, principals could explain the rationale, criteria, and intended objectives and provide formal channels for teacher feedback. Principals could also integrate aesthetic and meaning-related elements into existing instructional and professional learning activities rather than equating aesthetic leadership with campus decoration. For example, teacher meetings and professional learning activities could include the sharing and display of instructional improvements, reflective records, curriculum cases, and student work under clear and publicly available criteria. Research has linked organizational fairness and communication to employees’ evaluations of the organizational climate and has associated art, creative activities, and aesthetic experience in teacher learning with reflective discussion, professional agency, and changes in teaching practice (Albers et al., 2019; Anderson et al., 2022; Vveinhardt and Bendaraviciene, 2022).

Schools could further support teachers’ psychological empowerment by providing clearly defined forms of professional autonomy. Teacher groups could undertake small-scale instructional improvement projects, identify teaching problems, design solutions, collect classroom evidence, and share their findings in professional learning meetings. Principals could focus on providing resources, clarifying institutional boundaries, offering feedback, and responding to implementation difficulties rather than determining every step on behalf of teachers. Psychological empowerment comprises meaning, competence, self-determination, and impact and has been consistently associated with work engagement, positive work attitudes, and proactive behavior (Seibert et al., 2011; Spreitzer, 1995). Such arrangements are therefore consistent with teacher autonomy and professional agency, although their effectiveness should be evaluated rather than assumed.

Aesthetic leadership practices should also be grounded in fairness, respect, transparency, and psychological safety. Schools could publish the selection criteria for training, awards, curriculum projects, and professional development opportunities, provide confidential channels for reporting workplace conflict or inappropriate managerial behavior, and clarify administrative response procedures. Research in general education schools has identified management practices, administrative responsiveness, internal procedures, and the overall work climate as important conditions related to workplace mobbing prevention, while organizational research has linked favoritism, nepotism, communication, and procedural fairness to perceptions of organizational climate (Vveinhardt and Bendaraviciene, 2022; Vveinhardt and Jakucionyte, 2023). Symbolic expression, cultural activities, and environmental design should therefore complement rather than replace fair procedures and genuine teacher participation. Because these practices were not directly tested in the present study, schools could initially pilot them within a grade-level or subject-based group and evaluate their feasibility through teacher feedback, participation records, and implementation logs.

### 5.7 Limitations and future research

Despite the relatively clear findings, several limitations should be acknowledged. First, the study employed a cross-sectional, single-wave self-report design, which does not permit strong conclusions about causal or temporal relationships and may involve common-method variance. Future research could use longitudinal, experimental, or quasi-experimental designs, as well as multi-source or temporally separated data collection, to examine the proposed relationships more rigorously. Second, AMOS was used to assess the measurement models and the latent-variable mediation relationship, whereas the moderating effects and conditional indirect effects were examined using a unified conditional process model based on composite scale scores. This analytical strategy avoided treating latent-variable mediation coefficients and observed-variable moderation coefficients as directly comparable parameters within a single model. Nevertheless, conditional process analysis based on composite scores cannot fully account for measurement error. Future studies could employ latent-variable interaction modeling to estimate the direct, indirect, moderating, and conditional indirect associations simultaneously within a single structural equation model.

## 6 Conclusion

Using data from high school teachers in Hainan Province, China, this study examined the relationships among perceived principal aesthetic leadership (AL), teacher professional development (TPD), aesthetic experience (AE), and psychological empowerment (PE). The findings indicated that perceived principal AL was positively associated with TPD and that AE partially mediated this statistical relationship. PE also significantly moderated the direct association between AL and TPD, with the association becoming stronger at higher levels of PE. Overall, the study identified a statistical indirect association through aesthetic experience and a pattern of moderation involving psychological empowerment in the relationship between perceived principal aesthetic leadership and teacher professional development.

## Data Availability

The datasets presented in this study can be found in online repositories. The names of the repository/repositories and accession number(s) can be found at: https://github.com/zeheyin-code/cobe.
