# Supplementary material for: Perceived principals’ aesthetic leadership and teacher professional development: the roles of aesthetic experience and psychological empowerment among Chinese high school teachers
Source: Front Psychol. 2026 Sep 10;17:1846303. doi: 10.3389/fpsyg.2026.1846303 (PMC13601028; doi:10.3389/fpsyg.2026.1846303)
Supplement: Supplementary file 1 [file Data_Sheet_1.docx]

**Supplementary material**

Supplementary Table S1. List of expert reviewers.

| **Expert** | **Institution Type** | **Institution Name** | **Title** | **Academic Background** |
| --- | --- | --- | --- | --- |
| 1 | High school | A High School | Senior Secondary Teacher | Educational Management |
| 2 | Normal university | A Normal University | Professor | Education |
| 3 | High school | B High School | Senior Teacher | Education |
| 4 | Normal university | C Normal University | Associate Professor | Education |
| 5 | High school | D High School | Senior Teacher | Arts |
| 6 | Comprehensive university | E Vocational University | Professor | Educational Management |
| 7 | Normal university | F Normal College | Associate Professor | Philosophy |
| 8 | Industry association / research institute | G Education and Training | Senior Teacher | Arts |

Supplementary Table S2. Items revised or deleted based on content validity evaluation and item analysis.

| **Code** | **Original Item Content** | **Revised Content / Deletion** | **Reason** |
| --- | --- | --- | --- |
| AC1-5 | The principal knows under what circumstances and at what speed to express what kind of emotion. | Deleted | Deleted after expert review |
| AC1-6 | The principal’s aesthetic perspective is concrete and pragmatic. | The principal is concrete and pragmatic in communication. | - |
| AA2-2 | The principal is good at using conflict in aesthetic imagery. | In creation or practice, the principal intentionally uses conflict to achieve an aesthetic effect. | - |
| AA2-6 | The principal is a pioneer of aesthetic change. | Deleted | Did not meet the criterion |
| AA2-8 | The principal can express his or her uniqueness through personal demeanor. | Deleted | Deleted after expert review |
| AA2-5 | The principal can use his or her own aesthetic views to change the views of people around him or her. | The principal can use his or her own views to change the views of people around him or her. |  |
| AS3-6 | The principal is able to manage aesthetic feelings. | [Partially obscured in source image] | Did not meet the criterion |
| AS3-11 | The principal can prevent any … through appropriate behavior. | [Partially obscured in source image] | Deleted after expert review |
| AY4-1 | The principal has placed aesthetic materials in the open spaces of the school buildings. | The principal has placed aesthetic materials in appropriate locations in the school buildings. | - |
| AY4-2 | The principal has arranged aesthetically pleasing objects in the remaining spaces of the school. | Deleted | Did not meet the criterion |
| AY4-9 | The principal strives to break limitations and seeks better results in the school’s aesthetic arrangement. | Deleted | Did not meet the criterion |
| AW5-4 | The principal has created a cultural environment that awakens the school’s potential in aesthetics. | Deleted | Did not meet the criterion |
| AM7-3 | The principal’s physical characteristics leave a deep impression on others. | The principal’s physical characteristics leave a favorable aesthetic impression on others. | - |
| CM1-3 | I apply the teaching knowledge or theories I have read to my teaching. | Deleted | Did not meet the criterion |

Note 1. AC1-1 to AC1-7 are items under the Aesthetic Communication dimension; AA5-1 to AA5-7 are items under the Aesthetic Method dimension; AS3-1 to AS3-10 are items under the Aesthetic Support dimension; AY4-1 to AY4-11 are items under the Aesthetic Application dimension; AW5-1 to AW5-7 are items under the Aesthetic Sensitivity dimension; AH6-1 to AH6-3 are items under the Aesthetic Integrity dimension; and AM7-1 to AM7-3 are items under the Appearance Attractiveness dimension;CM1-3 is an item in the Teaching Content and Methods dimension of the Teacher Professional Development Scale.

Supplementary Table S3. Summary of confirmatory factor analysis for the perceived principals’ aesthetic leadership scale (N = 611).

| **Dimension** | **Item** | **Standardized Factor Loading** | **CR** | **AVE** |
| --- | --- | --- | --- | --- |
| Aesthetic Communication | AC1-1 | .922 | .925 | .642 |
|  | AC1-2 | .696 |  |  |
|  | AC1-3 | .918 |  |  |
|  | AC1-4 | .899 |  |  |
|  | AC1-5 | .641 |  |  |
|  | AC1-6 | .768 |  |  |
|  | AC1-7 | .713 |  |  |
| Aesthetic Method | AA2-1 | .773 | .924 | .660 |
|  | AA2-2 | .724 |  |  |
|  | AA2-3 | .833 |  |  |
|  | AA2-4 | .823 |  |  |
|  | AA2-5 | .935 |  |  |
|  | AA2-6 | .890 |  |  |
| Aesthetic Support | AS3-1 | .725 | .932 | .606 |
|  | AS3-2 | .880 |  |  |
|  | AS3-3 | .697 |  |  |
|  | AS3-4 | .712 |  |  |
|  | AS3-5 | .860 |  |  |
|  | AS3-6 | .790 |  |  |
|  | AS3-7 | .810 |  |  |
|  | AS3-8 | .734 |  |  |
|  | AS3-9 | .831 |  |  |
| Aesthetic Application | AY4-1 | .870 | .943 | .656 |
|  | AY4-2 | .763 |  |  |
|  | AY4-3 | .714 |  |  |
|  | AY4-4 | .781 |  |  |
|  | AY4-5 | .945 |  |  |
|  | AY4-6 | .892 |  |  |
|  | AY4-7 | .840 |  |  |
|  | AY4-8 | .789 |  |  |

Supplementary Table S3 (continued). Summary of confirmatory factor analysis for the perceived principals’ aesthetic leadership scale (N = 611).

| **Dimension** | **Item** | **Standardized Factor Loading** | | **CR** | **AVE** |
| --- | --- | --- | --- | --- | --- |
| Aesthetic Sensitivity | AW5-1 | | .762 | .931 | .688 |
|  | AW5-2 | | .864 |  |  |
|  | AW5-3 | | .747 |  |  |
|  | AW5-4 | | .928 |  |  |
|  | AW5-5 | | .943 |  |  |
|  | AW5-6 | | .797 |  |  |
| Aesthetic Integrity | AH6-1 | | .900 | .821 | .690 |
|  | AH6-2 | | .771 |  |  |
|  | AH6-3 | | .650 |  |  |
| Appearance Attractiveness | AM7-1 | | .799 | .826 | .614 |
|  | AM7-2 | | .855 |  |  |
|  | AM7-3 | | .689 |  |  |

**Note 1. AC1-1 to AC1-7 are items under the Aesthetic Communication dimension; AA5-1 to AA5-7 are items under the Aesthetic Method dimension; AS3-1 to AS3-10 are items under the Aesthetic Support dimension; AY4-1 to AY4-11 are items under the Aesthetic Application dimension; AW5-1 to AW5-7 are items under the Aesthetic Sensitivity dimension; AH6-1 to AH6-3 are items under the Aesthetic Integrity dimension; and AM7-1 to AM7-3 are items under the Appearance Attractiveness dimension.**

Supplementary Table S4. Summary of confirmatory factor analysis for the teacher professional development scale (N = 611).

| **Dimension** | **Item** | **Standardized Factor Loading** | **CR** | **AVE** |
| --- | --- | --- | --- | --- |
| **Continuous Updating of Teaching Content** | CM1-1 | .891 | .943 | .810 |
|  | CM1-2 | .888 |  |  |
|  | CM1-3 | .919 |  |  |
|  | CM1-4 | .897 |  |  |
| **Teaching Experimentation** | TE2-1 | .899 | .958 | .786 |
|  | TE2-2 | .915 |  |  |
|  | TE2-3 | .835 |  |  |
|  | TE2-4 | .970 |  |  |
|  | TE2-5 | .871 |  |  |
| **Reflection and Feedback Seeking** | RF3-1 | .685 | .943 | .759 |
|  | RF3-2 | .890 |  |  |
|  | RF3-3 | .942 |  |  |
|  | RF3-4 | .790 |  |  |
|  | RF3-5 | .926 |  |  |
| **Collaboration With Colleagues to Improve Classroom Teaching** | CI4-1 | .848 | .947 | .767 |
|  | CI4-2 | .918 |  |  |
|  | CI4-3 | .870 |  |  |
|  | CI4-4 | .922 |  |  |
|  | CI4-5 | .881 |  |  |
| **Collaboration With Colleagues to Promote School Development** | CD5-1 | .893 | .956 | .793 |
|  | CD5-2 | .911 |  |  |
|  | CD5-3 | .937 |  |  |
|  | CD5-4 | .776 |  |  |
|  | CD5-5 | .924 |  |  |

**Note 1.** CM1-1 to CM1-5 are items under the dimension of **Continuous Updating of Teaching Content**; TE2-1 to TE2-5 are items under the dimension of **Teaching Experimentation**; RF3-1 to RF3-5 are items under the dimension of **Reflection and Feedback Seeking**; CI4-1 to CI4-5 are items under the dimension of **Collaboration With Colleagues to Improve Classroom Teaching**; and CD5-1 to CD5-5 are items under the dimension of **Collaboration With Colleagues to Promote School Development**.

Supplementary Table S5. Summary of confirmatory factor analysis for the aesthetic experience scale (N = 611).

**(N = 611)**

| **Dimension** | **Item** | **Standardized Factor Loading** | **CR** | **AVE** |
| --- | --- | --- | --- | --- |
| **Pleasure in Beauty** | PB1-1 | .867 | .927 | .680 |
|  | PB1-2 | .825 |  |  |
|  | PB1-3 | .849 |  |  |
|  | PB1-4 | .799 |  |  |
|  | PB1-5 | .715 |  |  |
|  | PB1-6 | .881 |  |  |
| **Aesthetic Attitude** | AA2-1 | .759 | .914 | .673 |
|  | AA2-2 | .721 |  |  |
|  | AA2-3 | .847 |  |  |
|  | AA2-4 | .830 |  |  |
|  | AA2-5 | .931 |  |  |
| **Understanding of Beauty** | UB3-1 | .731 | .904 | .654 |
|  | UB3-2 | .843 |  |  |
|  | UB3-3 | .831 |  |  |
|  | UB3-4 | .828 |  |  |
|  | UB3-5 | .840 |  |  |
| **Complete Experience** | CE4-1 | .893 | .911 | .672 |
|  | CE4-2 | .843 |  |  |
|  | CE4-3 | .862 |  |  |
|  | CE4-4 | .764 |  |  |
|  | CE4-5 | .727 |  |  |

**Note 1.** PB1-1 to PB1-6 are items under the dimension of **Pleasure in Beauty**; AA2-1 to AA2-5 are items under the dimension of **Aesthetic Attitude**; UB3-1 to UB3-5 are items under the dimension of **Understanding of Beauty**; and CE4-1 to CE4-5 are items under the dimension of **Complete Experience**.

Supplementary Table S6. Summary of confirmatory factor analysis for the psychological empowerment scale (N = 611).

| **Dimension** | **Item** | **Standardized Factor Loading** | **CR** | **AVE** |
| --- | --- | --- | --- | --- |
| **Work Meaning** | S1-1 | .708 | .791 | .558 |
|  | S1-2 | .788 |  |  |
|  | S1-3 | .743 |  |  |
| **Self-Efficacy** | SE2-1 | .662 | .765 | .521 |
|  | SE2-2 | .744 |  |  |
|  | SE2-3 | .756 |  |  |
| **Self-Determination** | SD3-1 | .775 | .820 | .574 |
|  | SD3-2 | .762 |  |  |
|  | SD3-3 | .735 |  |  |
| **Work Impact** | I1-1 | .746 | .782 | .544 |
|  | I1-2 | .710 |  |  |
|  | I1-3 | .756 |  |  |

**Note 1. S1-1 to S1-3 are items under the dimension of Work Meaning; SE2-1 to SE2-3 are items under the dimension of Self-Efficacy; SD3-1 to SD3-3 are items under the dimension of Self-Determination; and I1-1 to I1-3 are items under the dimension of Work Impact.**
